# Effects of Adult Müller Cells and Their Conditioned Media on the Survival of Stem Cell-Derived Retinal Ganglion Cells

**DOI:** 10.3390/cells9081759

**Published:** 2020-07-22

**Authors:** Xandra Pereiro, Adam M. Miltner, Anna La Torre, Elena Vecino

**Affiliations:** 1 Department of Cell Biology and Histology, University of Basque Country UPV/EHU, Leioa, 48940 Vizcaya, Spain; xandra.pereiro@ehu.eus; 2 Department of Cell Biology and Human Anatomy, University of California Davis, Davis, CA 95616, USA; ammiltner@ucdavis.edu (A.M.M.); alatorre@ucdavis.edu (A.L.T.)

**Keywords:** retinal ganglion cells, Müller glia, Stem cells, retinal organoids, neuroprotection, neuritogenesis

## Abstract

Retinal neurons, particularly retinal ganglion cells (RGCs), are susceptible to the degenerative damage caused by different inherited conditions and environmental insults, leading to irreversible vision loss and, ultimately, blindness. Numerous strategies are being tested in different models of degeneration to restore vision and, in recent years, stem cell technologies have offered novel avenues to obtain donor cells for replacement therapies. To date, stem cell–based transplantation in the retina has been attempted as treatment for photoreceptor degeneration, but the same tools could potentially be applied to other retinal cell types, including RGCs. However, RGC-like cells are not an abundant cell type in stem cell–derived cultures and, often, these cells degenerate over time in vitro. To overcome this limitation, we have taken advantage of the neuroprotective properties of Müller glia (one of the main glial cell types in the retina) and we have examined whether Müller glia and the factors they secrete could promote RGC-like cell survival in organoid cultures. Accordingly, stem cell-derived RGC-like cells were co-cultured with adult Müller cells or Müller cell-conditioned media was added to the cultures. Remarkably, RGC-like cell survival was substantially enhanced in both culture conditions, and we also observed a significant increase in their neurite length. Interestingly, Atoh7, a transcription factor required for RGC development, was up-regulated in stem cell-derived organoids exposed to conditioned media, suggesting that Müller cells may also enhance the survival of retinal progenitors and/or postmitotic precursor cells. In conclusion, Müller cells and the factors they release promote organoid-derived RGC-like cell survival, neuritogenesis, and possibly neuronal maturation.

## 1. Introduction

Degenerative diseases of the retina are one of the main causes of irreversible vision loss. Notably, retinal ganglion cell (RGC) death is the common hallmark of several ocular conditions, including glaucoma, Leber′s hereditary optic neuropathy (LHON), and other optic neuropathies [1,2,3]. RGCs are the only output neurons of the retina as their axons form the optic nerve and connect the retina with the brain. As a result, RGC degeneration can lead to vision loss and, in fact, glaucoma is one of the leading causes of blindness worldwide [4]. Although RGC loss is the cornerstone in the management of glaucomatous degenerations, the only FDA-approved treatments are designed to lower intraocular pressure to slow disease progression. Unfortunately, these treatments do not reverse RGC damage and thus, fail to reverse vision loss. Therefore, the drive to develop strategies to functionally replace damaged RGCs has intensified in recent years [5,6,7].

Pluripotent stem cells are able to self-renew but these cells can also differentiate into any cell lineage of an adult organism, including all retinal cell populations [8,9,10]. Due to this potential, pluripotent cells offer an unlimited source of donor cells to replace damaged cells in retinal degenerations. In the last decade, the ability to generate retinal neurons from pluripotent stem cells using three-dimensional (3D) organoid cultures has become well established [10,11,12,13,14,15,16,17]. This method is based on plating dissociated pluripotent cells onto low-adhesion plates. Since the stem cells cannot attach to the bottom of the plate, they attach to each other, forming 3D aggregates called embryoid bodies (EBs) that subsequently develop into organoids with organized layers that contain all the normal retinal neurons. As this in vitro stem cell culture method mirrors normal retinal development, rod photoreceptors greatly outnumber the RGC-like cells produced, as naturally occurs in the mouse and human retina [18,19]. Indeed, the average yield of stem cell-derived RGC-like cells has been estimated to be between 0.1% and 30% of the total number of cells [15,20,21,22,23,24], similar to the normal percentage of RGCs in the retina at different developmental stages [25,26,27]. Additionally, the number of RGC-like cells decreases over time in 3D cultures [28,29]. This decline is not unexpected given that newly born RGCs undergo two waves of programmed cell death during normal development, as they become critically dependent on trophic support from their synaptic targets [30]. Since the brain targets of these cells (e.g., the lateral geniculate nucleus-LGN) is not present in organoid cultures, Bax-mediated apoptosis is likely to be responsible for the temporal decline in RGC-like cell number [31].

Müller cells are one of the principal glial cell populations in the retina, and they can enhance survival and neuritogenesis of RGCs in culture [32,33,34]. This support is preferentially due to direct physical interactions, although it has been demonstrated that Müller cell-conditioned medium (CM) can also significantly enhance the survival of cultured adult porcine RGCs [32]. Interestingly, some data indicates that the neuroprotection afforded by Müller cell CM exceeds whole retina CM [35]. Similarly, Müller cell-derived trophic factors appear to not only improve RGC survival but also promote RGC neuritogenesis [36,37,38].

Given the vast number of people who could benefit from RGC transplantation [4], it is essential to establish reliable and cost-effective methods to produce high numbers of healthy RGCs before their transplantation can be feasibly assessed in clinical trials. In an attempt to overcome the decline of stem cell-derived RGC-like cells obtained from pluripotent cells and in light of the neuroprotection afforded to RGCs by Müller cells, in this study we evaluate the effects of co-culturing stem cell-derived RGCs-like cells and adult Müller glia, and how glial cell CM influences survival and neuritogenesis.

## 2. Materials and Methods

### 2.1. Animals

Adult mice (*Mus musculus*, CD-1 IGS) were obtained from Charles River Laboratories (Wilmington, MA, USA). All the animals had ad libitum access to food and water, and were kept at a constant temperature of 21 °C on a 12 h light/12 h dark cycle. All mouse husbandry and handling was in accordance with protocols approved by the University of California Davis Animal Care and Use Committee (IACUC protocol #19413), which strictly adheres to all NIH guidelines and satisfies the Association for Research in Vision and Ophthalmology guidelines for animal use.

### 2.2. Mouse ESC Culture

Mouse embryonic stem cells (mESCs; R1 line) were obtained from ATCC (SCRC-1011, Manassas, VA, USA). The undifferentiated cells were maintained in medium containing leukemia inhibitory factor (LIF) and MAPK and GSK3β inhibitors (LIF + 2i): ES qualified Dulbecco′s modified Eagle′s medium (DMEM) supplemented with 20% heat-inactivated fetal bovine serum (FBS, Thermo Fisher, Carlsbad, CA, USA), non-essential amino acids (NEAAs) (Thermo Fisher, Waltham, MA, USA), sodium pyruvate (Thermo Fisher, Waltham, MA, USA), 0.1 mM β-mercaptoethanol (Sigma Aldrich, Saint Louis, MO, USA), 100 µL of LIF (10 million units/mL: ESGRO, Millipore, Billerica, MA, USA), 3 µM of a GSK3β inhibitor (Stemgent, Cambridge, MA, USA), and 0.4 µM of a MEK inhibitor (Stemgent). All undifferentiated cells were maintained in feeder-free conditions using growth factor-reduced Matrigel-coated plates (GFR-Matrigel was from Corning, New York, NY, USA). Only low-passage (< passage 30) cultures were used for these experiments.

### 2.3. Retinal Differentiation of mESCs

Retinal differentiation of mESCs was performed following a previously defined protocol, with minor variations [9]. Semi-confluent undifferentiated colonies were dissociated to a single cell suspension using TrypLE (Gibco, Rockville, MD, USA) and gentle mechanical dissociation. Subsequently, 5000 undifferentiated cells were plated in 96-well ultra-low attachment plates (Sbio, Hudson, NH) in retinal differentiation (RD) medium (Day 0): Glasgow minimum essential medium (GMEM; Thermo Fisher) supplemented with 1% NEAAs, 1% sodium pyruvate, 1.5% Knock-Out Serum Replacement (KSR; Thermo Fisher), and 0.01 mM β-mercaptoethanol (Sigma-Aldrich). In these conditions, free-floating mESCs spontaneously formed aggregates (EBs) in less than 12–14 h. Twenty four hours later (Day 1), 2% Growth Factor Reduced-Matrigel (Corning) was added to each well. On day 4, the EBs were moved to low-attachment six-well plates (Thermo Fisher) and the medium was replaced with 3:1 RD media and Tom′s medium. The formulation of Tom’s medium is as follows: Neurobasal A medium (Thermo Fisher), 1% Bovine serum albumin (BSA; Invitrogen, Eugene, OR, USA), 1% B27, 1% N2, 1% NEAAs, 0.4% HEPES, 1% sodium pyruvate, and 0.075% sodium bicarbonate (all from Thermo Fisher). On day 5, the medium was replaced with to 1:1 RD medium:Tom’s medium; and on day 6, the medium was replaced with 1:3 RD medium:Tom′s medium. Finally, on day 7, the medium was replaced with 100% Tom’s medium and the EBs were maintained until day 10–15, as indicated in each experiment.

### 2.4. Adult Müller Cell Cultures

Mice were sacrificed by cervical dislocation and their eyes were enucleated. The corneas, crystalline, lens, and the vitreous bodies were removed, and the retinas were carefully extracted in fresh DMEM/-CO_2_ medium. The retinas were then cut into small fragments and they were incubated at 37 °C for 30 min in a Sterile Earle’s Balanced Salt Solution (EBSS) containing Papain (20 U/mL) and DNase (2000 U/mL: Worthington, Lakewood, NJ, USA). Enzyme digestion was stopped by adding Ovomucoid, according to manufacturer’s instructions, and the tissue was then mechanically dissociated. Isolated cells were recovered by centrifugation at 1200 rpm for 5 min. The pelleted cells were resuspended in DMEM + 10% FBS, seeded onto sterile 13 mm glass coverslips in 24 well plates, coated with poly-l-lysine (100 µg/mL: Sigma-Aldrich) and laminin (10 µg/mL: Sigma-Aldrich), and further cultured in DMEM + 10% FBS. All the cells recovered from one mouse retina were seeded in one well. The cultures were maintained in a humidified incubator at 37 °C in an atmosphere of 5% CO_2_. The media was replaced with fresh media on day 1 of culture and subsequently, every 3 days. These cultures reached confluence after 7 days in vitro (DIV).

### 2.5. Co-Cultures of Stem Cell-Derived RGC-Like Cells and Müller Cells.

Day 10–15, EBs were collected and mechanically dissociated with Accutase (Stem Cell Technologies), centrifuged at 1200 rpm for 2 min and resuspended in DMEM + 10% FBS. Dissociated EBs were seeded on a monolayer of Müller cells and maintained for 3 days in one of three different media: (1) Neurobasal supplemented with B27 and N2; (2) Neurobasal supplemented with B27, N2, and 10% FBS; or (3) DMEM supplemented with 10% FBS. Dissociated EBs seeded onto sterile poly-l-lysine and Laminin-coated 13 mm glass coverslips in 24 well plates were used as controls.

### 2.6. Müller Cell Conditioned Media Collection

Conditioned media (CM) was collected when Müller cell cultures had reached confluence (day 7), first washing the wells three times with DMEM medium supplemented with 1% l-glutamine and 0.1% gentamicin (Thermo-Fisher Waltham, MA, USA). DMEM was then added to each well and left for 3 h before the medium was changed to eliminate the rest of the FBS. Fresh DMEM was then added for 2 days before it was collected and sterilized by passing through a 0.22 μm filter. The CM was frozen in aliquots at −20 °C, and the Müller cells that produced it were fixed for 10 min with methanol at −20 °C.

### 2.7. Application of Conditioned Media

EBs dissociated on day 10 were plated (10 EBS/well) on 13-mm poly-l-lysine and Laminin coated glass coverslips in 24-well plates to test the activity of the CM. The cultures were maintained in a mixture of 50% Neurobasal medium supplemented with 2% B27 and N2, 1% l-glutamine (Thermo-Fisher) and 0.1% gentamicin, and 50% CM. The cells were cultured for 3 days at 37 °C in a humidified atmosphere containing 5% CO_2_, and the medium was changed every 2 days. Cells maintained in Neurobasal medium supplemented with 2% B27 and 1% l-glutamine were used as controls. The cells were fixed for 10 min with ice-cold methanol at −20 °C on day 3.

Undissociated day 10 EBs were also maintained in a mixture of 50% Tom’s and 50% CM for 3 days, using EBs maintained in Tom´s medium alone as controls. The EBs were then collected for RNA extraction and at least three replicates of each culture were made, performing the procedure in triplicate.

### 2.8. Immunocytochemistry

After 3 days in co-culture or in the presence of CM, the cells were fixed in cold methanol (−20 °C) and washed with PBS (pH 7.0). After blocking non-specific antigens with blocking buffer (10% NGS and 0.1% Triton X-100 in PBS), the cells were incubated with primary antibodies at a dilution indicated in Table 1 at 4 °C overnight. The next day, the cells were extensively washed with PBS and incubated with Alexa Fluor secondary antibodies as indicated in Table 1. Subsequently, the cells were washed with PBS, the cell nuclei were labeled with DAPI at a dilution of 1:10,000, and the coverslips were then mounted with Fluoromount-G (Southern Biotech, Birmingham, AL, USA).

### 2.9. qPCR

Total RNA was extracted from the whole EBs maintained in CM using Trizol (Invitrogen) and chloroform extraction, according to the manufacturer′s instructions. The RNA was digested with DNase1 (Qiagen, Hilden, Germany), cleaned using the Qiagen RNA mini clean-up kit and reverse transcribed into cDNA using the Superscript III RT kit (Invitrogen) following the manufacturer′s instructions. qPCR was performed using the primers indicated in Table 2.

### 2.10. Quantification and Statistical Analysis of RGC-Like Cells

Fluorescent images of RGC-like cells and Müller cells were taken on a Leica DM 5000 M fluorescence microscope with a Leica DFC 500 camera using the same exposure times to compare control and treatment conditions. The total number of RGC-like cells per coverslip was quantified using the specific markers indicated for each experiment. The RGC-like cells and Müller cells in co-cultures were counted, and statistical analyses were carried out using the IBM SPSS Statistics software v.21-0 and the homogeneity of the variances was assayed with the Levene′s test. A Mann–Whitney U test or ANOVA were used to assess whether there were significant differences between the groups. The minimum value of significance for both tests was defined as *p* < 0.05. At least four complete coverslips and three independent experiments were analyzed for each experimental condition.

## 3. Results

### 3.1. Mouse Embryonic Stem Cells can be Differentiated into RGC-Like Cell Fates

**Figure 1:**
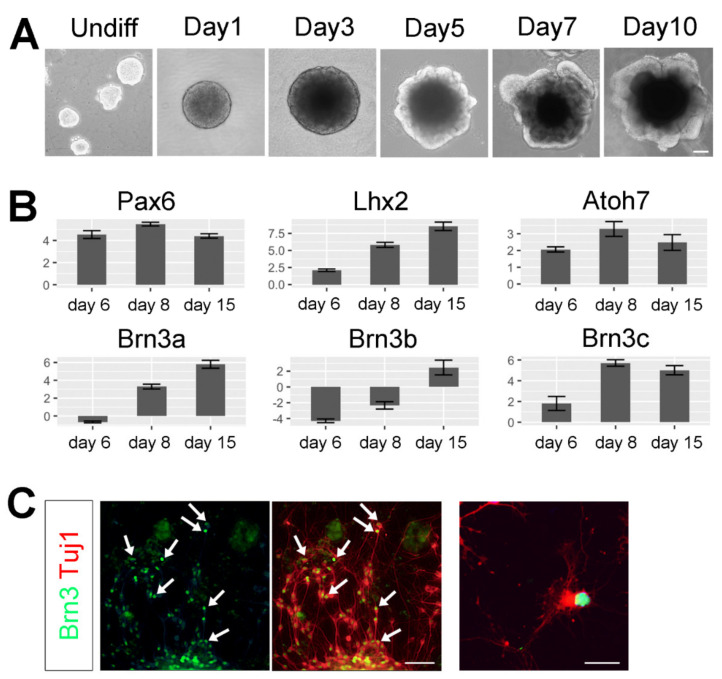
Typical morphologies of embryoid bodies (EBs) at the different stages of differentiation. (**A**) Bright field images of the different differentiation stages of EBs from day 0 to day 10, Scale bar: 100 µm, (**B**) RT-qPCR analyses. *Y*-axis represents fold difference compared to undifferentiated control samples and error bars represent standard errors. All data was normalized to beta-Actin (ddCT). (**C**) Day 13 EBs were dissociated and plated onto coated coverslips, and labeled with β-III-tubulin (red) and Pan-Brn3 (green) antibodies. Scale bar: 100 µm (left panel) and 50 µm (right panel).

R1 undifferentiated mouse embryonic stem cells (mESCs) were directed towards retinal cell fates following a protocol originally described by Yoshiki Sasai’s laboratory [12], with some modifications from our previously described method [39]. Upon differentiation, free-floating mESCs formed EBs that developed a neural epithelium that was apparent by day 3 as a well-defined clear layer in the outer part of each EB (Figure 1A). By day 5, the neuroepithelial layer evaginated and formed optic vesicle-like structures. These optic regions increased in size and thickness in the subsequent culturing days (Figure 1A). To further confirm that the mESCs were directed towards retinal cell fates, we performed RT-qPCR analyses. At day 6, several retinal progenitor genes (Pax6, Lhx2 and Atoh7) were highly upregulated compared to the undifferentiated samples (Figure 1B, upper panels). Atoh7/Math5 is normally expressed in a subpopulation of retinal progenitor cells in the developing retina and is required for RGC and optic nerve development in mouse and humans [40,41,42]. Thus, the observed expression in the organoid cultures suggests that the retinal progenitors in the EBs acquired the competence to generate RGC-like cells at these early differentiation stages.

By day 8, a significant increase in the expression of the RGC-specific genes Brn3a (Pou4f1), Brn3b (Pou4f2) and Brn3c (Pou4f3) was detected (Figure 1B, lower panels). Stem cell-derived RGC-like cells were dissociated from EBs cultured for 10−15 days and, as shown in Figure 1C, a pan-Brn3 antibody abundantly co-labeled β-III-tubulin+ neurons resembling RGCs (white arrows in Figure 1C).

### 3.2. Adult Mouse Müller Glia Cells Cultured for 7 Days Express Normal Müller Glia Markers

**Figure 2:**
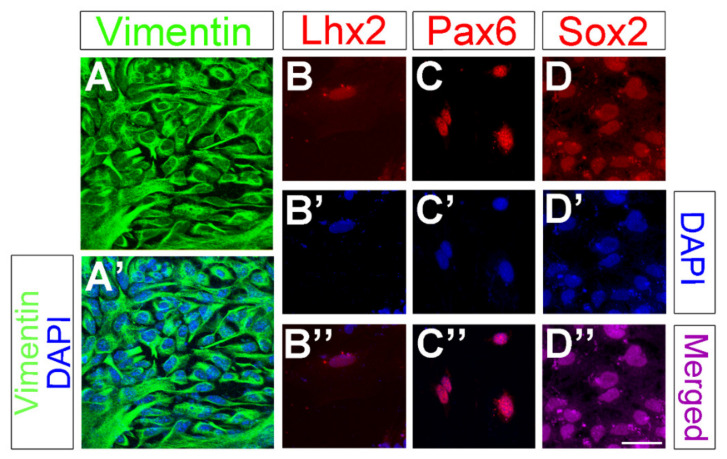
Cultured Müller glia cells express normal Müller glia markers. Vimentin (**A**, **A**’, green), Lhx2 (**B**, **B**’’, red), Pax6 (**C**, **C**’’, red), Sox2 (**D**, **D**’’, purple), and DAPI (**A**’, **B**’, **B**’’, **C**’, **C**’’, **D**’, **D**’’, blue) expression in Müller glia cell cultures. Scale bar: 50µm.

We have recently established a method to purify and culture adult Müller glia cells from mouse, rat and pig retinas, and we have previously shown that cultured murine Müller glia cells express GFAP, Glutamine Synthetase (GS), p75^NTR^, and Cralbp. Flow cytometry indicated that >94% of all the cells in these cultures express p75^NTR^ [43]. In order to further characterize our Müller glia culture system, we used a combination of markers normally expressed in Müller glia cells, including the cytoskeletal protein Vimentin (Figure 2A,A’, green) and the transcription factors Lhx2, Pax6, and Sox2 (Figure 2B–D, red). Negative controls for each secondary antibody employed were also performed in order to verify the specificity of the primary antibodies. As expected, cultured Müller glia cells expressed high levels of Vimentin, and variable but detectable levels of Lhx2, Pax6 and Sox2 were observed in Müller glia nuclei.

### 3.3. Co-Culturing Stem Cell-Derived Cells with Adult Müller Glia Highly Increased RGC-Like Cell Survival

**Figure 3:**
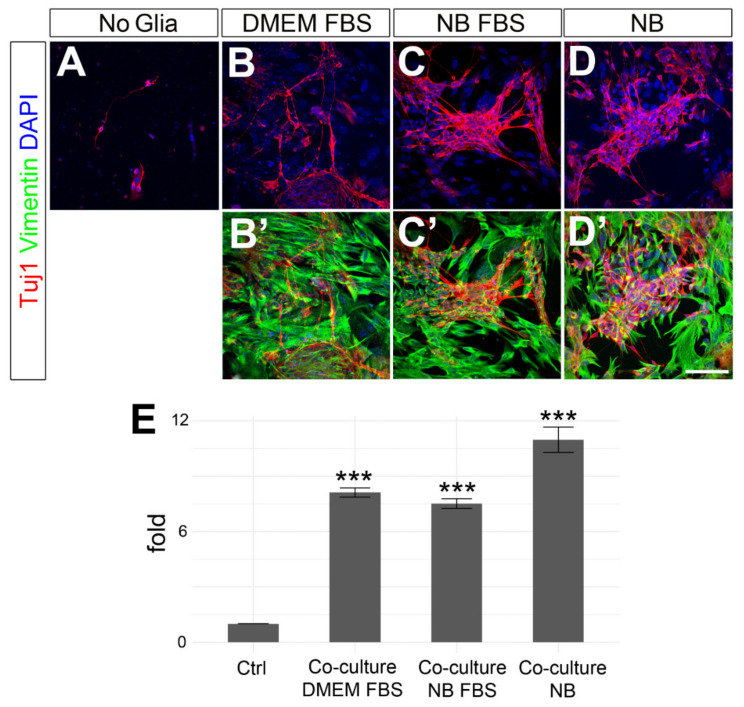
Effects of co-culturing stem cell-derived cells with Müller glia on RGC-like cell survival. Images of stem cell-derived RGC-like cells in (**A**) control conditions or (**B–D’**) co-cultured with adult Müller cells. The cells were labeled with antibodies against β-III-tubulin (red) for RGCs, Vimentin (green) for Müller cells and nuclei were co-stained with DAPI (blue). (**E**) Quantification of RGC survival (fold change relative to controls) in the presence of Müller cells. The cells were cultured either in DMEM supplemented with 10% FBS; Neurobasal medium (NB) supplemented with N2, B27 and 10% FBS; or Neurobasal medium (NB) supplemented with N2 and B27 only. Statistical significance was established by one-way ANOVA with Tukey post-hoc test. *p*-values: *** < 0.001. Scale bar: 100 µm.

In order to assess the possible neuroprotective effects of Müller glia on stem cell-derived RGC-like cells, dissociated EBs were co-cultured on a monolayer of adult mouse Müller cells for 3 days in three different media: Neurobasal medium supplemented with B27 and N2 with or without 10% FBS (more favorable to neurons), and DMEM + 10% FBS (more favorable to glia, Figure 3). Neurobasal is a medium optimized for neuronal cell culture [44], and the use of Neurobasal instead of DMEM has been shown to be essential for good survival of purified RGCs [45]. Similarly, our previous data indicated that Müller glia cells grow better when cultured in DMEM supplemented with 10% FBS. Given that the ideal culturing conditions for RGCs and Müller glia are different, we tested different media compositions to address what is the best culturing condition to support RGC survival when neurons and glia are combined in a co-culture system. Interestingly, when stem cell-derived cells were grown on Müller cells, RGC-like cell survival was enhanced in all the different media tested in comparison to the control conditions (the absence of Müller cells). On average, RGC-like cell survival increased 8.11 fold ± 0.25 (mean ± std. deviation) when the cells were cultured in DMEM + 10% FBS (*p* = 0.00086), 7.5 fold ± 0.26 (mean ± std. deviation) when cultured in Neurobasal + B27 + N2 + 10% FBS (*p* = 0.000109), and 10.97 fold ± 0.68 (mean ± std. deviation) when cultured in Neurobasal with B27 and N2 (*p* = 0.00001, Figure 3E). While we detected clear differences between the control cells and the co-cultures, there were no statistical differences between the different culture conditions assayed (DMEM vs. Neurobasal + 10% FBS *p* = 0.3482, DMEM vs. Neurobasal *p* = 0.2392, Neurobasal vs. Neurobasal + 10% FBS *p* = 0.86).

### 3.4. Müller Glia Conditioned Media (CM) Enhances RGC-Like Cell Survival

**Figure 4:**
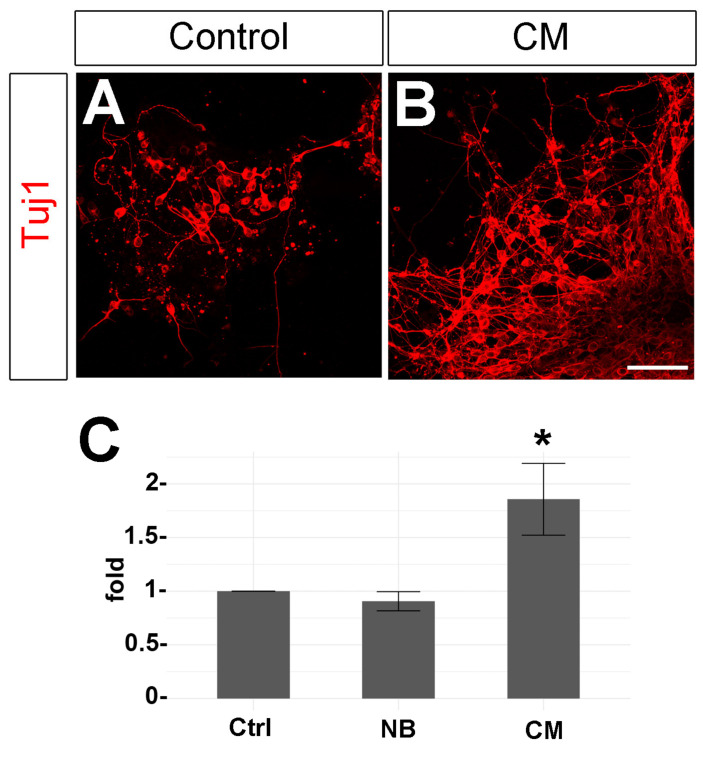
Effects of Müller cell-conditioned medium (CM) on stem cell-derived RGC-like cells survival in vitro. Images of stem cell-derived RGC-like cells maintained in (**A**) control conditions or (**B**) cultured with adult Müller cell CM. The cells were labeled with antibodies against β-III-tubulin (red). (**C**) Quantification of RGC survival (fold change relative to controls) in the presence of CM. Statistical significance was established by one-way ANOVA with Tukey post-hoc test. *p*-value: * < 0.05. Scale bar: 50 µm.

To analyze if the factors secreted by adult Müller cells have the same effect on the survival of stem cell-derived RGC-like cells similar to the co-cultures, dissociated EBs were cultured with adult mouse Müller cell CM. RGC-like cells cultured in DMEM + 10% FBS (control) were compared to those maintained in Neurobasal medium supplemented with B27 and N2 (Neurobasal control) or to cells maintained in 1:1 Neurobasal medium B27:Müller cell CM. Notably, we observed significantly more RGC-like cells when the cells were maintained in the presence of Müller cell CM. Exposing the cultured stem cell-derived cells to the Müller cell CM enhanced the survival of RGC-like cells 1.85 ± 0.33 folds (mean ± std. deviation, *p*-value: 0.00174), while there were no significant differences between DMEM and Neurobasal culturing conditions (*p*-value: 0.715, Figure 4).

### 3.5. Müller Glia Conditioned Media (CM) Increases Atoh7 and Brn3b Expression

**Figure 5:**
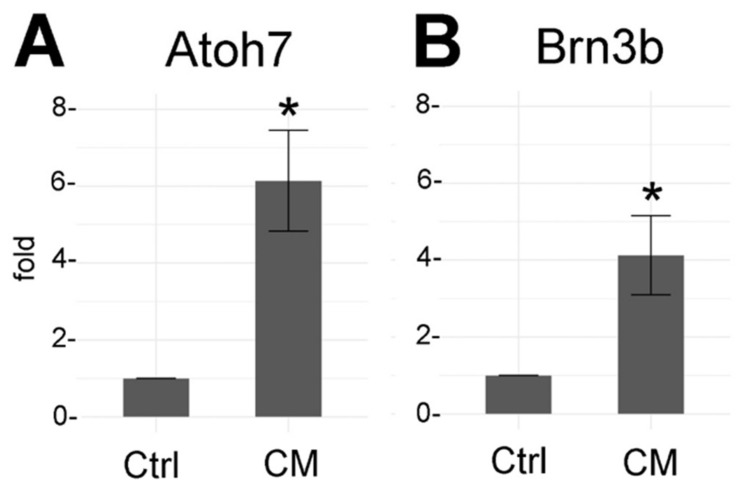
Brn3b and Atoh7 expression in EBs cultured in adult Müller cell-conditioned media (CM). Relative Brn3b (**A**) and Atoh7 (**B**) expression in EBs maintained in Müller cell CM. Data shown as fold change compared to control cells and normalized to Beta-Actin (ddCT). Student’s *t*-test was used to compare control and experimental condition for statistical significance. *p*-value: * < 0.05.

The effect of the Müller cell CM on the survival of stem cell-derived RGC-like cells was also assessed in non-dissociated EBs by analyzing the expression of Brn3b and Atoh7 by RT-qPCR. Brn3b (Pou4f2) is a protein expressed in most RGCs [46,47,48,49,50], while Atoh7 is a transcription factor that is transiently expressed during early retinal histogenesis and is necessary for RGC development [40,51,52]. Both Brn3b and Atoh7 expression were enhanced when EBs were cultured with adult Müller cell CM; Atoh7 was upregulated 6.14-fold (*p* = 0.0309) and Brn3b 4.12-fold (*p* = 0.021, Figure 5).

### 3.6. Co-Culturing RGC-Like Cells with Müller Glia Cells as Well as Müller Glia Conditioned Media (CM) Increases Neuritogenesis

**Figure 6:**
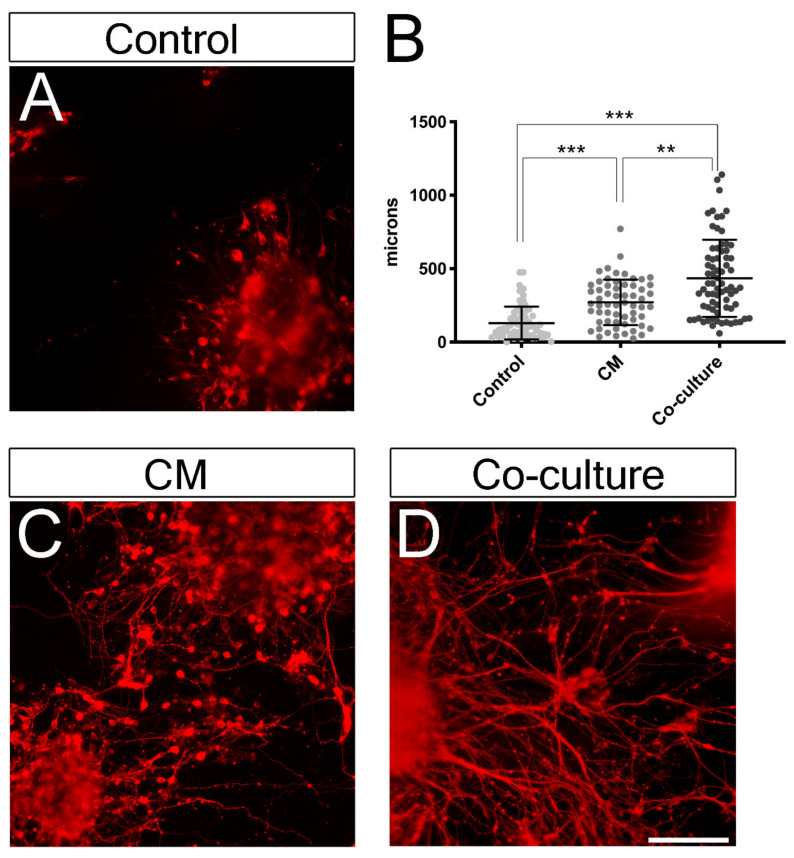
Effects of Müller cell co-cultures and conditioned medium (CM) on stem cell-derived RGC-like cell neuritogenesis. Images of stem cell-derived RGC-like cells maintained in (**A**) control conditions or (**C**) cultured in Müller cell CM or (**D**) co-cultured with adult Müller cells. The cells were labeled with antibodies against β-III-tubulin (red). (**B**) Quantification of RGC-like cell neurite length. Statistical significance was established by one-way ANOVA with Tukey post-hoc multiple comparison test. *p*-value: ** < 0.005, *** < 0.001. Scale bar: 100 µm.

Previous data have shown that Müller glia can enhance neuritogenesis of cultured adult RGCs [32,37]. To assess whether a similar effect was also elicited on stem cell-derived RGC-like cells, we quantified the neurite length of the longest neurite of cells cultured in control conditions (DMEM + 10% FBS), cells exposed to CM (1:1, DMEM + 10% FBS: CM), and RGC-like cells co-cultured with Müller glia. To avoid including dying cells in the quantifications, all experiments were co-stained with DAPI and we excluded from the counts any cells exhibiting pyknotic nuclei or axonal fragmentation. Remarkably, both CM and co-culturing conditions significantly increased neurite length (Figure 6). At least, 70 individual neurites were measured from three different biological replicates for each condition. In control conditions, the longest neurite grew 129.7 ± 112 μm (mean ± std. deviation); the cells exposed to Müller glia-conditioned media extended neurites that measured 270.6 ± 155 μm (mean ± std. deviation), while the RGCs co-cultured with Müller glia exhibited significantly longer neurites that grew 434.3 ± 262 μm (mean ± std. deviation), with the longest neurites measuring over 1 mm.

## 4. Discussion

In recent years, stem cell therapies have become a promising strategy to develop novel treatments for retinal degenerations. As stem cell protocols have advanced, derivation methods and utilization of specific culturing conditions have been established to set the proper conditions for controlled and directed differentiation towards retinal cell fates, including RGC-like cells [11,15,20,39,53,54]. Our data indicates that, in culture, organoids acquire the competence to generate RGC-like cells at early stages of differentiation as retinal progenitor genes are upregulated from day 6 (Figure 1B) and RGC-associated genes (Brn3a, b and c) are increased from day 8, mimicking the normal developmental progression of the embryonic retina [55,56,57]. Surprisingly, Brn3b initially exhibits a decline in expression (day 6 in vitro, Figure 1B). While most of the commonly used retinal markers are not expressed in undifferentiated stem cells, it has been previously shown that Brn3b is expressed in germ cells and stem cells at the earliest stages of embryonic development [58], and thus, the initial decline probably reflects the ongoing differentiation from Brn3b+ undifferentiated cells.

Stem cell-derived RGC-like cells have been previously characterized by their molecular signatures and physiological properties [23,24,59,60]. These experiments validate that the cells made from organoids in vitro are remarkably similar to endogenous RGCs even though differences in their maturation status have been reported [61]. Correspondingly, retinal organoids yield different RGCs subtypes, can fire action potentials, and respond to normal chemoattractant and chemorepellent cues [23,62,63]. However, within 3D retinal organoid cultures, RGC-like cells die over time [29] and, in fact, these cells are not observed in mouse retinal organoids beyond differentiation day 25 [64]. This currently unavoidable cell loss is likely the result of two main factors: RGC-like cell number in culture is probably governed by the same programmed cell death processes that occur during normal development [65,66], and a failure to locate brain targets and establish normal synaptic connections in vitro. Another factor driving RGC-like cell degeneration in culture may be a technical limitation: Organoids grown in free-floating 3D cultures have limited access to oxygen and nutrients. This is in stark contrast to a living animal with constant blood flow to replenish all tissues and cells with nutrients and oxygen. Regardless of the molecular causes of the degeneration, novel strategies must be devised to increase organoid-derived RGC survival before we can translate these technologies to clinical applications.

Glia are typically considered the support cells of the nervous system due to their critical functions in providing structural and metabolic support, regulating the blood-brain barrier, and controlling synaptic modulation and tissue homeostasis [67]. One of the most abundant glial types in the retina, the Müller glia, expands across all the layers of the retina and it has been well-characterized that these cells can enhance the survival of different retinal neurons [68]. Importantly, Müller glia functions have been shown to increase survival of RGCs [32,37,69]. For instance, Müller glia secrete neurotrophic factors like BDNF [70], CTNF [71], basic fibroblast growth factor (bFGF) [72], pigment epithelium-derived growth factor (PEDGF) [73], and glial-derived neurotrophic factor (GDNF) [74]. Classic experiments have shown that these trophic factors can promote RGC survival in vitro [36,75,76] and in experimental models of RGC damage [77,78,79]. In addition to these roles, Müller glia are also known to phagocytose cell debris in physiological and pathological conditions [80,81,82]. Given the strong neuroprotective effect of Müller glia on neurons, we have developed a strategy to use adult Müller cells to enhance the survival of stem cell-derived RGC-like cells during retinal differentiation.

Notably, we have shown that co-culturing RGC-like cells derived from stem cells together with Müller glia greatly enhances survival and neuritogenesis. Similarly, dissociated EBs cultures were maintained with medium conditioned by Müller cells to assess whether the increase of organoid-derived RGC-like cell survival was due to factors released by the Müller cells. The conditioned medium also significantly increased the survival and neuritogenesis (Figure 4 and Figure 6), indicating that the effect of Müller cells is not mediated by substrate alone as we have previously observed [32,38]. However, as the effects of co-culturing cells on the survival of stem cell-derived RGC-like cells were greater than that observed with CM alone, there appears to be a synergistic effect of membrane bound and diffusible factors. It is also possible that the half-life of factors necessary to significantly improve the health of stem cell-derived RGCs is short, and the presence of Müller glia that constantly replenish these factors increases the beneficial effect these factors have on RGCs in vitro. Additionally, Müller glia cells can elicit phagocytic functions in coordination with the microglia, often considered the “professional” phagocytic cells of the retina [83,84,85]. During development, the microglia originates in the primitive yolk sac [86] and infiltrate in the retina during early development. Since they have a different embryonic origin, microglia cells are not present in stem cell-derived retinal organoids as the stem cells are first directed towards ectodermal fates. Importantly, it has been suggested that the phagocytic activity of Müller cells becomes more relevant in degenerative situations where the microglia are absent [87]. By clearing debris and removing cellular corpses, Müller glia may prevent subsequent damage to other neurons in stem cell-derived cultures.

In a series of elegant experiments, the Meyer lab has recently shown that co-culturing hiPCS-derived RGC-like cells with astrocytes, another important glial type of the retina, increased RGC neurite length and complexity, and improved RGC maturation, including a significant enhancement of the RGC electrophysiological properties [61]. Overall, the neurite length in control conditions measured in this study as well as the effect elicited by co-culturing with astrocytes are comparable to our results even though we are using mouse cells. In contrast, astrocyte-conditioned media showed no effects in RGC neuritogenesis. In our experiments, Müller glia CM not only increased neuritogenesis but also enhanced the expression of Brn3b in non-dissociated EBs, suggesting an enhanced survival of stem cell-derived RGCs in non-dissociated EBs (Figure 5), similar to the survival detected in isolated cells. We also assessed the expression of Atoh7, a transcription factor that is transiently expressed during early retinal histogenesis and that is necessary for RGC differentiation [40,52] or survival [88]. Our results show that Atoh7 expression is enhanced in EBs grown in CM, suggesting that the factors released by Müller cells not only enhance the survival of RGCs but also they increase the survival of the progenitor cells that can differentiate into RGCs.

In conclusion, Müller cells release factors that enhance the survival of stem cell-derived RGC-like cells and also contribute to neuritogenesis and maturation. These findings could improve stem cell-based transplant strategies currently under development. In future studies, the role of Müller glia should be considered when developing in vitro models using RGCs. Moreover, given the important role of Müller glia in RGC survival, future studies should also shed light on the molecular relationships between the Müller glia and the RGCs in pathological conditions, perhaps opening novel targets for therapeutic strategies for the treatment of glaucoma and other RGC degenerations.

## Figures and Tables

**Table 1:** List of antibodies used in this study.

Antibody	Specificity	Catalogue	Dilution	Source
Pan-Brn3	RGCs	sc-6026	1:200	Santa Cruz, Dallas, TX, USA
β-III-tubulin	RGCs	801201	1:1000	Biolegend, San Diego, CA, USA
Vimentin	Müller glia	N/A	1:1000	gift from Dr. P. FitzGerald
Lhx2	Müller glia	sc-19344	1:250	Santa Cruz, Dallas, TX, USA
Pax6	Müller glia	901301	1:200	Biolegend, San Diego, CA, USA
Sox2	Müller glia	sc-17320	1:500	Santa Cruz, Dallas, TX, USA
Alexa 488 anti-goat	Goat IgG	A11055	1:500	Thermo Fisher, Carlsbad, CA, USA
Alexa 568 anti-mouse	Mouse IgG	A10037	1:500	Thermo Fisher, Carlsbad, CA, USA
Alexa 488 anti-rabbit	Rabbit IgG	A21206	1:500	Thermo Fisher, Carlsbad, CA, USA

**Table 2:** List of qPCR primers (5′ to 3′).

Primers	Sequence
Pou4f1 Forward	5′-CGC GCA GCG TGA GAA AAT G-3′
Pou4f1 Reverse	5′-CGG GGT TGT ACG GCA AAA T-3′
Pou4f2 Forward	5′-CGT ACC ACA CGA TGA ACA GC-3′
Pou4f2 Reverse	5′-AGG AGA TGT GGT CCA GCA GA-3′
Pou4f3 Forward	5′-CGA CGC CAC CTA CCA TAC C-3′
Pou4f3 Reverse	5′-CCC TGA TGT ACC GCG TGA T-3′
Atoh7 Forward	5′-CCC TAA ATT TGG GCA AGT GAA GA-3′
Atoh7 Reverse	5′-CAA AGC AAC TCA CGT GCA ATC-3′
Lhx2 Forward	5′-CTG TTC CAG AGT CTG TCG GG-3′
Lhx2 Reverse	5′-CAG CAG GTA GTA GCG GTC AG-3′
Pax6 Forward	5′-CTG GAG AAA GAG TTT GAG AGG-3′
Pax6 Reverse	5′-TGA TAG GAA TGT GAC TAG GAG-3′
B-Actin Forward	5′-CTA AGG CCA ACC GTG AAA AG-3′
B-Actin Reverse	5′-ACC AGA GGC ATA GAG GGA CA-3′
GAPDH Forward	5′-TGA CCA GAG TCC ATG CCA TC-3′
GAPDH Reverse	5′-GAC GGA CAC ATT GGG GGT AG-3′
